# Impact of PDCA cycle-driven nutritional support on serum biomarkers and quality of life in nasopharyngeal carcinoma patients undergoing radiotherapy

**DOI:** 10.5937/jomb0-56797

**Published:** 2025-11-05

**Authors:** Weiming Xiong, Jinying Mo, Jingjin Weng, Yongli Wang, Jiazhang Wei, Linsong Ye, Min Li, Shenhong Qu

**Affiliations:** 1 Jinan University, Guangzhou, China; 2 Department of Otolaryngology, Guangxi Zhuang Autonomous Region People's Hospital, Guangxi Academy of Medical Sciences, Nanning, China

**Keywords:** PDCA cycle management, NPC, radiotherapy, nutritional support, blood nutritional biomarkers, QoL, PDCA ciklus upravljanja, NPC, radioterapija, nutritivna podrška, nutritivni biomarkeri u krvi, kvalitet života

## Abstract

**Background:**

This study aimed to assess the effectiveness of nutritional support guided by the PDCA (Plan-Do-Check-Act) cycle management model in nasopharyngeal carcinoma (NPC) patients undergoing radiotherapy.

**Methods:**

A total of 100 NPC patients between December 2021 and October 2023 were randomly assigned to two groups: an observation group (OG, n = 50) and a control group (CG, n = 50). The CG received routine nutritional support, while the OG received nutritional support based on the PDCA cycle. Key outcomes included blood nutritional biomarkers, quality of life (QoL), incidence of adverse reactions (ARs), and clinical efficacy.

**Results:**

Post-treatment analysis showed that the OG had a significantly higher total effective rate (72%) compared to the CG (38%) (P&lt; 0.05). The OG also showed higher albumin, prealbumin, and total protein levels and lower CRP levels after treatment. The OG significantly improved cognitive, role, social, physical, and emotional functioning (P&lt; 0.05). The AR rate was considerably lower in the OG (20%) compared to the CG (36%) (P&lt; 0.05).

**Conclusions:**

These results suggest that PDCA cycle-based nutritional support enhances clinical efficacy and QoL, reduces nutritional risks and adverse reactions and improves overall safety in NPC patients undergoing radiotherapy. Serum markers like serum iron, ferritin, prealbumin, and CRP effectively monitored the impact of nutritional interventions on patients' nutritional and inflammatory status.

## Abstract (sr)

**Uvod:**

Ova studija imala je za cilj da proceni efikasnost nutritivne podrske vođene PDCA (Plan-Do-Check-Act) ciklusom upravljanja kod pacijenata sa karcinomom nazo-farinksa (NPC) na radioterapiji.

**Metode:**

Ukupno 100 pacijenata sa NPC, lečenih između decembra 2021. i oktobra 2023, nasumično je raspore-đeno u dve grupe: posmatranu grupu (OG, n = 50) i kontrolnu grupu (CG, n = 50). CG je primala standardnu nutritivnu podršku, dok je OG dobijala nutritivnu podršku zasnovanu na PDCA ciklusu. Ključni ishodi uključivali su nutritivne biomarkere u krvi, kvalitet života (QoL), učestalost neželjenih reakcija (NR) i kliničku efikasnost.

**Rezultati:**

Analiza nakon tretmana pokazala je da OG ima značajno višu ukupnu stopu efikasnosti (72%) u poređenju sa CG (38%) (P&lt; 0,05). Takođe, OG je imala više nivoe albumina, prealbumina i ukupnih proteina, kao i niže nivoe CRP-a nakon terapije. Kognitivna, socijalna, fizička, emocionalna i uloga životne funkcionalnosti su značajno poboljšane u OG (P&lt; 0,05). Stopa NR bila je znatno niža u OG (20%) u poređenju sa CG (36%) (P&lt; 0,05).

**Zaključak:**

Ovi rezultati sugerišu da nutritivna podrška zasnovana na PDCA ciklusu poboljšava kliničku efikasnost i kvalitet života, smanjuje nutritivne rizike i neželjene reakcije te povećava ukupnu bezbednost kod pacijenata sa NPC na radioterapiji. Serumski markeri poput serumskog gvožđa, feritina, prealbumina i CRP-a efikasno prate uticaj nutritivnih intervencija na nutritivni i inflamatorni status pacijenata.

## Introduction

Nasopharyngeal carcinoma (NPC) is a malignant head and neck tumour with a relatively high malignancy. Most pathological types of NPC are poorly differentiated or undifferentiated squamous cell carcinomas. Due to its hidden and deep location in the nasopharynx, early detection is challenging. Additionally, early symptoms of NPC are often non-specific, leading to frequent misdiagnosis or missed diagnoses [1] [2] [3]. Common initial presentations of NPC include nasal bleeding, epistaxis, tinnitus, hearing loss, nasal congestion, headache, and visual disturbances, which can progress to blindness, visual field defects, diplopia, exophthalmos, restricted movement, neuroparalytic keratitis, cranial nerve damage, and cachexia.

Advances in medical technology have significantly improved NPC outcomes, with an overall cure rate of 80% across all stages and a five-year survival rate of up to 98% for stage I NPC. However, recurrence rates remain high [4] [5]. Anatomically, the nasopharynx resembles a round-arch hexagon with six interconnected walls. Tumour invasion in any region can lead to widespread lesions due to its proximity to critical structures such as the skull base, cranial nerves, cervical spine, pharyngeal muscles, and fascia [6] [7] [8]. The lymphatic network in the nasopharynx is exceptionally dense and connected, which can lead to cancer spreading to lymph nodes on both sides of the neck, even if the primary tumour is only on one side.

Surgical treatment for NPC is challenging due to its complex anatomy, but most NPC cases involve poorly differentiated squamous cell carcinoma, which is highly radiosensitive [9]. As a result, radiotherapy remains the first-line treatment. Traditionally, NPC has been treated with radiotherapy and chemotherapy, followed by consolidation chemotherapy to enhance efficacy. The typical regimen involves 1.5 months of chemoradiotherapy followed by two to three courses of consolidation chemotherapy. Given the 30-40% risk of distant metastasis, radiotherapy plays a pivotal role in reducing the spread of cancer cells [10] [11] [12].

Clinical nutrition focuses on understanding nutrients' nature, distribution, metabolic effects, and the consequences of inadequate food intake [13]. Nutritional support, introduced in the 1950s, involves providing comprehensive nutrients to patients via oral, gastrointestinal, or parenteral routes [14] [15] [16]. Over 30% of hospitalised patients require nutritional support, which has been recognised as a significant medical achievement alongside antibiotics, anaesthesia, intensive care, and organ transplantation [17] [18]. A well-balanced nutritional plan can enhance the body's disease resistance, improve surgical and anaesthetic outcomes, strengthen immunity, promote recovery, reduce postoperative complications, lower medical costs, and shorten hospital stays [19]. An appropriate and standardised nutritional support strategy is critical for ensuring optimal outcomes in clinical care.

Nutritional support is vital for NPC patients undergoing radiotherapy. Serum markers such as prealbumin, C-reactive protein (CRP), serum iron, and ferritin have been shown to reflect both nutritional status and the inflammatory response to treatment. Prealbumin, a short-term marker of nutritional status, helps assess immediate changes in protein synthesis during treatment. Elevated CRP levels are indicative of systemic inflammation and may correlate with radiotherapy-induced tissue damage and complications. Iron and ferritin levels are essential for evaluating iron deficiency and anaemia, common complications in cancer treatment, and the effects of iron on the overall quality of life (QoL) and efficacy of therapy. Monitoring these markers with routine nutritional indices provides a comprehensive picture of the patient's nutritional and immune status, aiding in personalised treatment planning.

This study enrolled 100 NPC patients treated with radiotherapy at Guangxi Zhuang Autonomous Region People's Hospital, Guangxi Academy of Medical Sciences, Nanning, China, between December 2021 and October 2023. Patients were randomly assigned to an observation group (OG) and a control group (CG), with 50 participants each. The CG received routine nutritional support, while the OG received nutritional support guided by the PDCA cycle management model. The Plan-Do-Check-Act (PDCA) cycle is a systematic, iterative methodology for quality management and continuous improvement. It involves four stages: planning specific objectives and strategies (Plan), implementing the proposed plan (Do), assessing the outcomes (Check), and making necessary adjustments based on the evaluation (Act). Widely applied in healthcare, the PDCA model enhances efficiency and consistency, making it a valuable tool for optimising clinical practices, including nutritional support in cancer care. The study compared blood nutritional biochemical indices, including Prealbumin, CRP Serum Iron, and Ferritin, as well as quality of life (QoL), incidence of adverse reactions (ARs), and clinical efficacy between the two groups to evaluate the effectiveness of PDCA-based nutritional support for NPC patients undergoing radiotherapy.

## Materials and methods

### Subjects

A total of 100 patients with nasopharyngeal carcinoma (NPC) undergoing radiotherapy at The Affiliated Hospital of Guangxi Zhuang Autonomous Region People's Hospital, Guangxi Academy of Medical Sciences, Nanning, China, between December 2021 and October 2023, were enrolled in this study. The cohort consisted of 56 men and 44 women, aged 40 to 78 years, with a mean age of 62.17±5.22 years. All participants provided written informed consent, with consent from their family members when applicable. The study received ethical approval from the Hospital Ethics Committee.

### Inclusion criteria:

- No prior surgery, radiotherapy, or chemotherapy.
- Availability of complete clinical data.
- Age ≥ 18 years.
- Willingness and ability to cooperate with follow-up assessments.

### Exclusion criteria:

- Pregnant women.
- Diagnosis of other malignant tumours.
- Severe wasting diseases.
- Distant metastasis.
- Serious organic diseases (e.g., heart, liver, or kidney disorders).

### Radiotherapy methods

Radiotherapy was performed with patients in the supine position, and a CT scan was taken from the skull to the clavicle. The images were transferred to a workstation for processing, and the target volume, including the nasopharynx, skull base, and neck, was delineated. Treatment was delivered using a Siemens linear accelerator at a dose of 1.5 Gy per session, administered once daily, five days per week, for a total dose of 75 Gy.

### Treatment methods

One hundred patients were randomly divided into two groups:

1. Control Group (CG): This group received routine nutritional support without dietary restrictions. No dietitian was involved in creating a structured plan. Patients were allowed to eat freely, and standard care was provided.
2. Observation Group (OG): The OG received nutritional support combined with a Plan-Do-Check-Act (PDCA) cycle approach. A dedicated PDCA management team comprises attending physicians, dietitians, and nurses with over five years of clinical experience. This team developed a tailored nutritional support plan based on each patient's activity levels, psychological conditions, and other factors.

- ° *Nutritional Support:* Dietitians and medical staff guided patients to maintain a balanced diet. Alternative feeding methods, such as nasogastric or abdominal enterostomy tubes, were used for those unable to eat orally.
- ° *PDCA Cycle Management:* The PDCA cycle involves four stages:

- *Plan:* Develop a nutritional support plan.
- *Do:* Implementation of the plan with individual patient care.
- *Check:* Regular monitoring of nutritional indicators.
- *Act:* Adjustments to the treatment plan based on evaluation results.

Nurses conducted weekly checks to assess the implementation, identify barriers to goal achievement, and provide rewards for compliance. This iterative process continued until patients fully adhered to the dietary recommendations.

### Observation indicators

*1. Comparison of Clinical Efficacy:* The clinical efficacy of the groups was evaluated using the following categories:

- *° Complete Remission (CR):* Relief and control of NPC symptoms with no evidence of spread.
- *° Partial Remission (PR):* Improvement of NPC symptoms without spread.
- *° Stable Disease (SD):* No progression or spread of NPC symptoms.
- *° Disease Progression (DP):* Worsening of NPC symptoms with evidence of spread.

The effective rate was calculated based on these categories.

*2. Blood Sample Analysis:* Venous blood samples were collected from each patient in the early morning before and after radiotherapy. The following biomarkers were measured for analysis:

- ° Serum Iron
- ° Ferritin
- ° Prealbumin
- ° C-reactive protein (CRP)
- ° Albumin
- ° Total Protein
- ° Lymphocyte Count

These blood indicators were analysed to assess the nutritional and inflammatory status of the patients.

*3. Anxiety Assessment:* The Self-Rating Anxiety Scale (SAS) was used to assess patient anxiety levels over the past week. The scale was unaffected by age, gender, or economic status, although patients with low education levels or cognitive impairments were excluded from self-assessment.

° Scores were rated on a 4-point scale:

- 1 = None
- 2 = A little
- 3 = Considerable
- 4 = Most of the time

° Anxiety levels were categorised as follows:

- Normal: Below 49
- Mild Anxiety: 50-59
- Moderate Anxiety: 60-69
- Severe Anxiety: Above 69

*4. Depression Assessment:* Depression severity was assessed using the Self-Rating Depression Scale (SDS).

° A 4-point scale was used:

- 1 = None
- 2 = A little
- 3 = Considerable
- 4 = Most of the time

° Severity was categorised as:

- Mild Depression: 53-62
- Moderate Depression: 63-72
- Severe Depression: Above 72

*Quality of Life (QoL) Assessment:* QoL was measured using a clinical scale covering five dimensions: cognitive function, role function, social function, physical function, and emotional function. Higher scores indicated better QoL outcomes.

*Adverse Event Monitoring:* Adverse events (AEs) were recorded and monitored, including anaemia, skin reactions, oral mucosal damage, nausea, vomiting, and thrombocytopenia. The adverse reaction (AR) rate was calculated for both groups.

### Routine nutritional support in the control group

The Control Group (CG) in this study received routine nutritional support focused on basic nutritional care without a structured or personalised dietary plan. Patients were not provided with a specific nutritional regimen tailored to their needs. Instead, the standard care followed general guidelines for nutrition during radiotherapy. There were no specific dietary restrictions, and patients were encouraged to maintain their usual dietary habits, free to choose what they ate. Notably, there was no involvement of dietitians in developing individualised meal plans or assessing the nutritional needs of each patient. This routine support ensured that patients received basic nutritional guidance during radiotherapy treatments. Still, it did not include specialised interventions or adjustments based on the patient's activity level, psychological condition, or specific treatment requirements. Essentially, it served as a baseline care approach against which the intervention of the Observation Group (OG), which received tailored nutritional support via the PDCA cycle, was compared.

### Statistical methods

Statistical analysis was conducted using SPSS version 19.0. Data were presented as mean±standard deviation (mean±SD) for continuous variables, while categorical variables were expressed as percentages (%). Repeated measures analysis of variance (ANOVA) was performed to compare the relevant indicators between the two groups. Statistical significance was determined using a two-tailed test, with a p-value of less than 0.05 considered statistically significant.

## Results

### Comparison of clinical data of patients

**Figure 1:**
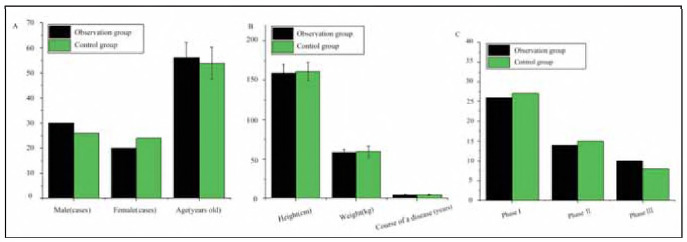
Demographic and clinical baseline data comparison between the observation group (OG) and control group (CG).<br>(A is the ratio of male to female, age; B, course of disease, height, and weight; C is clinical stage)

As shown in Figure 1, there were no significant differences between the two groups regarding gender ratio, age, disease duration, height, weight, or clinical stage (P>0.05).

### Comparison of clinical efficacy of patients

**Figure 2:**
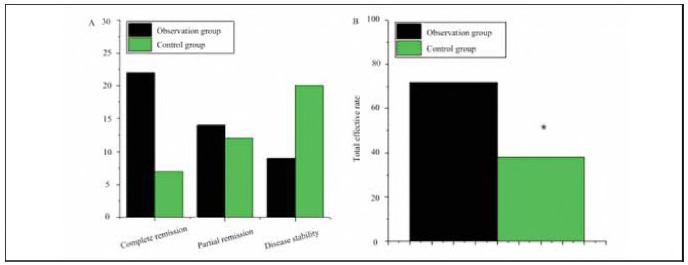
Comparison of clinical efficacy between OG and CG.<br>(A is complete response, partial response, stable disease, progressive disease; B is the total effective rate of treatment) Note: * indicates OG versus CG, P<0.05

In OG (CG), 22 (7) cases had complete remission, 14 (12) cases had partial remission, 9 (20) cases had stable disease, and 5 (11) cases had progressive disease, with a total effective rate of 72% (38%). The total treatment response rate was higher in OG (*P*<0.05) (Figure 2).

### Comparison of blood nutritional biochemical indexes

**Figure 3:**
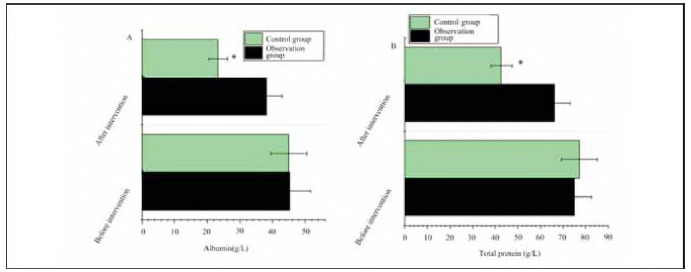
Comparison of albumin and total protein levels between OG and CG.

In OG, the albumin level before treatment was 45.28±6.44 g/L, and the total protein level was 75.06±7.65 g/L; after treatment, the albumin level was 38.07±4.72 g/L, and the total protein level was 66.23±7.11 g/L; in Controls, there was 44.95±5.58 g/L and 77.22±8.13 g/L, and 23.25±2.91 g/L and 42.75±4.68 g/L. There was a similarity in albumin and total protein levels between OG and CG before treatment (*P*>0.05). Albumin levels and total protein levels were higher in OG after treatment (*P*<0.05) (Figure 3).

**Figure 4:**
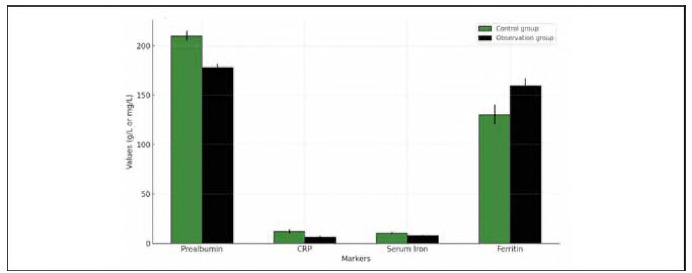
Comparison of serum iron, ferritin, prealbumin, and CRP levels before and after treatment

Additionally, serum iron, ferritin, prealbumin, and CRP levels were measured in both groups. In the OG, before treatment, the serum iron level was 10.52±2.08 μmol/L, ferritin was 125.36±15.28 ng/mL, prealbumin was 217.41±28.56 mg/L, and CRP was 11.02±3.46 mg/L. After treatment, serum iron decreased to 7.82±1.91 μmol/L, ferritin increased to 159.43±18.45 ng/mL, prealbumin decreased to 178.22±23.31 mg/L, and CRP decreased to 6.25±2.89 mg/L. In the CG, before treatment, serum iron was 10.74±2.15 μmol/L, ferritin was 130.29±16.02 ng/mL, prealbumin was 210.31 ±25.18 mg/L, and CRP was 12.37±3.72 mg/L. After treatment, serum iron decreased to 5.25±1.89 μmol/L, ferritin increased to 145.82±17.19 ng/mL, prealbumin decreased to 163.01 ±21.72 mg/L, and CRP increased to 9.53±3.56 mg/L. There were no significant differences in the serum iron and ferritin levels between the groups before treatment (P>0.05). However, after treatment, serum iron was significantly lower in the CG (P<0.05), ferritin was significantly higher in the OG (P<0.05), and CRP was considerably lower in the OG (P<0.05). Prealbumin levels remained significantly higher in the OG compared to the CG after treatment (P<0.05) (Figure 4).

**Figure 5:**
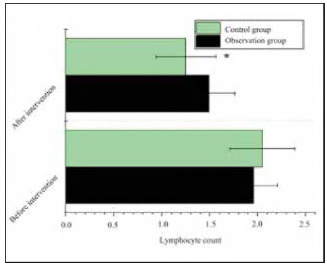
Comparison of lymphocyte counts before and after treatment.

The lymphocyte count of OG was 1.96 ±0.2 5 before treatment and 1.49±0.27 after treatment; The lymphocyte count of CG was 2.05±0.34 and 1.25±0.31, respectively. OG and CG had a similar lymphocyte count before treatment (P>0.05). The lymphocyte count after treatment was lower in OG (*P*<0.05) (Figure 5).

### Comparison of QoL

**Figure 6:**
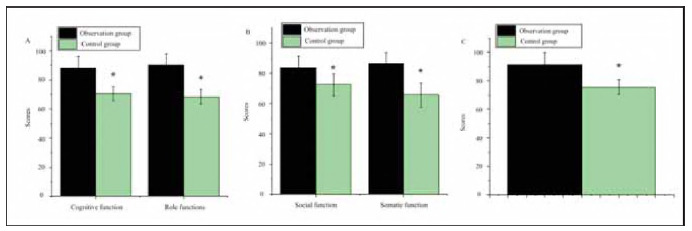
Comparison of quality of life (QoL) between OG and CG.<br>(A: cognitive function, role function; B: social function, physical function; C: emotional function)

As shown in Figure 6, after treatment, the OG group demonstrated the following mean scores: cognitive function (88.21 ±7.94), role function (90.33±8.05), social function (83.65±7.55), physical function (86.37±7.04), and emotional function (91.25±8.45). In contrast, the CG group exhibited significantly lower scores: cognitive function (70.58±4.85), role function (68.34±5.11), social function (72.36±7.33), physical function (65.52±8.04), and emotional function (75.42±5.21). The OG group showed superior outcomes across all function dimensions than the CG group (P<0.05).

### Comparison of ARs

**Figure 7:**
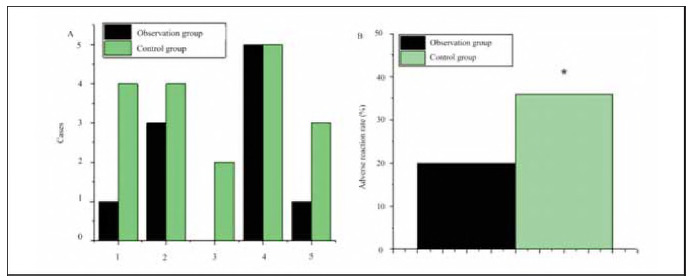
Comparison of adverse reactions (ARs) between OG and CG.<br>(A: skin reaction, anaemia, oral mucosal damage, nausea and vomiting, thrombocytopenia; B: AR rate)

As illustrated in Figure 7, the occurrence of adverse reactions (ARs) in the OG group included 1 case of skin reaction, 3 cases of anaemia, 0 cases of oral mucosal damage, 5 cases of nausea and vomiting, and 1 case of thrombocytopenia. In comparison, the CG group reported 4 cases of skin reaction, 4 cases of anaemia, 2 cases of oral mucosal damage, 5 cases of nausea and vomiting, and 3 cases of thrombocytopenia. The overall AR rate in the OG group was significantly lower (20%) compared to the CG group (36%) (P<0.05).

## Discussion

Nasopharyngeal carcinoma (NPC) is the most common malignant tumour among head and neck squamous cell carcinomas. It is particularly prevalent in southern China and Southeast Asia, closely associated with Epstein-Barr virus (EBV) infection [20]. Given the high radiosensitivity of most NPC tumours, radiotherapy remains the primary treatment modality for NPC. However, the extended duration and high dosage of radiotherapy often result in complications such as acneiform rashes, oral mucositis, radiation dermatitis, and trismus [21] [22]. Furthermore, dysphagia frequently induces anxiety in patients, underscoring the critical importance of appropriate nutritional support during and after radiotherapy. Malnutrition, resulting from treatment-related side effects and poor oral intake, exacerbates treatment toxicity and hampers recovery. It is essential to provide tailored nutritional interventions to maintain or improve patients' nutritional status.

The Plan-Do-Check-Act (PDCA) cycle, a widely recognised quality management methodology, divides the process into four stages: planning, execution, assessment, and action [23]. By continuously reviewing and improving practices, the PDCA cycle ensures ongoing optimisation of care processes. Its application in the medical field has substantially improved treatment quality and efficiency, particularly in managing complex diseases such as cancer [24]. In the present study, 100 NPC patients were randomly assigned to either the observation group (OG), which received nutritional support guided by the PDCA cycle, or the control group (CG), which received routine nutritional care. The study found no significant differences between the groups regarding clinical baseline characteristics, such as gender, age, disease duration, height, weight, or clinical stage (P>0.05), which ensured that the groups were comparable at the outset of the intervention.

Evaluating the effectiveness of novel treatment protocols can be challenging, especially in the oncology setting. In China, a four-level assessment system is commonly used to categorise outcomes as cure, marked effect, progress, or ineffectiveness [25]. The results of this study showed that the total effective rate of treatment in the OG (72%) was significantly higher than that in the CG (38%) (P<0.05), indicating that PDCA cycle-driven nutritional support significantly enhanced clinical efficacy in NPC patients undergoing radiotherapy.

One of the primary aims of nutritional support in cancer care is to improve key nutritional biomarkers. To assess patients' nutritional and inflammatory status, this study measured several serum markers, including albumin, total protein, serum iron, ferritin, prealbumin, and C-reactive protein (CRP). Post-treatment analysis showed that the OG had significantly higher albumin and total protein levels than the CG (P<0.05). Albumin, a major plasma protein, is essential in maintaining osmotic pressure and supporting immune function. Decreases in albumin levels during cancer treatment can indicate nutritional deficiencies and a higher risk of complications [26]. The OG also showed improvements in prealbumin levels, which, being a short-term marker of nutritional status, provides a sensitive indication of recent changes in nutrition. These results suggest that PDCA cycle-based nutritional support helps stabilise protein synthesis and improves nutritional status during radiotherapy.

It is important to consider its potential mechanisms on protein synthesis, inflammation, and immune function to understand how PDCA-based nutritional support improves outcomes. The PDCA cycle emphasises continuous monitoring and adjustment, which can optimise nutritional interventions that stabilise protein synthesis, such as increasing albumin and prealbumin levels. These proteins are essential for immune function and tissue integrity, which are compromised during radiotherapy. Furthermore, PDCA-based interventions help modulate inflammation by regulating markers such as C-reactive protein (CRP) and ferritin, reducing systemic inflammation and tissue damage. This holistic approach may improve immune function and overall recovery during cancer treatment.

Additionally, we assessed the serum iron and ferritin levels, critical indicators of iron metabolism. Serum iron levels were significantly lower in the OG after treatment, indicating reduced iron availability, likely due to the adverse effects of radiotherapy and treatment-related inflammation. In contrast, ferritin levels, a marker of iron storage, significantly increased in the OG, suggesting a potential compensatory response to iron depletion or inflammatory response. Ferritin is also an acute-phase reactant, and its elevation may reflect inflammation or tissue damage, which aligns with the observed decrease in CRP levels in the OG (P<0.05). CRP is a well-established marker of systemic inflammation and tissue damage, and a reduction in CRP levels in the OG suggests that PDCA-based nutritional support may help modulate the inflammatory response to radiotherapy [27].

Tang and colleagues explored the impact of nutritional intervention on nasopharyngeal carcinoma (NPC) patients undergoing radiotherapy and chemotherapy. Their findings show that nutritional support significantly improved patients' nutritional status, with lower NRS scores and better weight maintenance in the intervention group compared to the control group. The intervention group also reported higher quality of life (QoL) scores, particularly in physical and emotional aspects. These results highlight the crucial role of nutritional intervention in reducing malnutrition, improving treatment tolerance, and enhancing overall QoL in NPC patients, supporting its integration into standard cancer care.

A key finding of the present study was the significant improvement in quality of life (QoL) among the OG patients compared to the CG. After treatment, the OG showed superior outcomes across all dimensions of QoL, including cognitive function, role function, social function, physical function, and emotional function (P<0.05). These results align with previous studies that emphasise the positive impact of nutritional interventions on cancer patients' QoL [27]. Nutritional support not only improves physical functioning but also reduces the psychological burden, helping patients better cope with the side effects of treatment.

Furthermore, the adverse reaction (AR) rate was significantly lower in the OG (20%) compared to the CG (36%) (P<0.05). This suggests that PDCA cycle-based nutritional support enhances clinical efficacy and QoL and minimises treatment-related complications. The reduction in adverse reactions may be attributed to improved nutritional status, better immune function, and reduced inflammation, as indicated by the changes in CRP and prealbumin levels. These findings underscore the importance of a structured nutritional approach in optimising cancer treatment's overall safety and effectiveness.

## Conclusion

The results of this study demonstrate that PDCA cycle-based nutritional support significantly improves clinical outcomes in NPC patients undergoing radiotherapy. By enhancing key serum nutritional biomarkers, improving QoL, and reducing adverse reactions, this intervention offers a promising approach to managing the nutritional and inflammatory challenges associated with cancer treatment. Serum markers such as serum iron, ferritin, prealbumin, and CRP proved valuable in assessing the impact of nutritional support on the patients' nutritional and immune status. Future studies with larger sample sizes and more extended follow-up periods will be necessary to validate the effectiveness of this approach and its long-term benefits in cancer care further.

## Dodatak

### Acknowledgements

The authors would like to thank the staff and patients of The Affiliated Hospital of Guangxi Zhuang Autonomous Region People's Hospital, Guangxi Academy of Medical Sciences, Nanning, China for Nationalities for their invaluable contributions to this study - special thanks to the radiology and nutrition departments for their support during data collection and patient management.

### Funding

The Guangxi Science and Technology Base and Talent Project supported this research (GuiKe-AD20297069).

### Supplementary material

Supplementary data, including detailed methodology and additional statistical analyses, are available upon request from the corresponding author.

### Authors' contribution

Weiming Xiong and Jinying Mo contributed equally to the manuscript.

### Conflict of interest statement

All the authors declare that they have no conflict of interest in this work.
